# Content, Behaviour Change Techniques, and Quality of Postpartum Depression Apps to Be Recommended by Midwives: Systematic Search and Evaluation

**DOI:** 10.3390/nursrep14030170

**Published:** 2024-09-06

**Authors:** Amalia Ureña-Lorenzo, Maria del Mar Fernandez-Alvarez, Judit Cachero-Rodríguez, Ruben Martin-Payo

**Affiliations:** 1 Public Health Service of Principado de Asturias, 33001 Asturias, Spain; amaliaure97@gmail.com; 2 Faculty of Medicine and Health Sciences, Universidad de Oviedo, 33006 Oviedo, Spain; cacherojudit@uniovi.es (J.C.-R.); martinruben@uniovi.es (R.M.-P.); 3 PRECAM Research Group, Health Research Institute of Asturias (ISPA), 33011 Oviedo, Spain

**Keywords:** postpartum depression, mobile health, health promotion

## Abstract

Background: Postpartum depression is a public health problem that affects a considerable percentage of women. Despite the proliferation of related apps, there are limited data available on the best apps to prevent postpartum depression. We identified which apps available in Spanish could be recommended by midwives based on their content, quality, and behaviour change techniques, as a complementary tool for preventing postpartum depression in women. Methods: A systematic search was performed to identify apps available on iOS App Store and Google Play, which were used to replicate how patients’ access “postpartum depression prevention” apps. Apps’ quality, behaviour change potential, and contents were assessed. Results: A total of 1408 apps were identified, of which 7 were retrieved for assessment (0.5%). The mean objective and subject quality were 3.1 (SD = 1.01) and 2.7 (SD = 1.27), respectively. A total of 24 topics were identified. The mean ABACUS score was 6.6 (SD = 3.64), and the mean number of topics addressed by the apps was 9.9 (SD = 5.90). Conclusion: The results of the present study suggest that a specific free app is not available in Spanish for the prevention of postpartum depression, and only a small percentage of free apps should be recommended based on their quality, BCTs, and contents. The systematic review protocol was not registered.

## 1. Introduction

Postpartum depression is defined as a highly vulnerable phase characterized by episodes of depressive symptoms that occur within one year after delivery [1]. According to the World Health Organization [2], about 10% and 13% of women experience a mental disorder—primarily depression—during pregnancy and the immediate postpartum period, respectively. According to the literature, the prevalence of postpartum depression ranges from 14% to 25% [3]. More specifically, 30.3%, 26.0%, and 25.3% of women have postpartum depression at 2 months, 6 months, and 1 year postpartum, respectively, in Spain [4].

Motherhood itself is a period of vulnerability to mental health problems due to maternal physical health needs, baby care tasks, and changes in social relationships [5]. Postpartum depression should be considered as multi-factorial. There are some physical, behavioural, social, and living factors associated with a higher risk: excessive gestational weight gain and emotional eating [6], low dairy product intake [7], perceived stress [8], smoking; alcohol consumption [3,9], insomnia and poor sleep quality [10], and low social support [3,11] are positively associated with postpartum depression. In contrast, physical activity [9], family support, and social connections with significant others [11] are negatively associated with postpartum depression.

Untreated postpartum depression may negatively affect mothers’ mental and physical health [12]. In addition, children can also suffer the consequences of postpartum depression, specifically a decrease in the quality of care, which affects key aspects, such as breastfeeding, and a deterioration in the mother–child relationship, which can negatively affect the development of the early child’s communication and language skills and other behaviour disorders during childhood to adolescence [2,12]; therefore, it should be considered a priority public health problem [13].

It is essential to highlight that there is evidence of the effectiveness of prenatal interventions in preventing postpartum depression [14].

Currently, face-to-face interventions to prevent the health problem addressed are hardly available in healthcare systems. Even if such services were offered, some barriers could remain, such as inequity or difficulty in accessing health services. The literature suggests the use of mHealth tools to solve these barriers [15]. The use of mobile applications (apps) as a complement to usual face-to-face care [16,17] has been demonstrated to help prevent postpartum depression. Some of the advantages of these apps include their cost-effectiveness, the availability of customized options [18], and acceptance by pregnant and postpartum women as a complementary tool for monitoring mental health during pregnancy [18], collecting information about postpartum depression [19], or monitoring mood symptoms [20].

The effectiveness of these apps has also attracted the attention of researchers. The systematic review developed by Miura et al. [21] revealed that the use of apps improves the score on the Edinburgh Postpartum Depression Scale [22]. Among its conclusions, two important aspects stand out: on the one hand, the benefit of using apps to prevent this health problem when it is impossible to receive face-to-face care and, on the other hand, that the effectiveness of these apps improves when they include an automated component through psychosocial interventions. In addition, a pilot randomized research study was developed by Qin et al. [23], who developed a program based on psychoeducation and cognitive restructuring, through the use of CareMom app. They showed that those women who were part of the intervention group, that is, users of the app, showed acceptability towards its use, a significant decrease in the score of the Edinburgh Postnatal Depression Scale [22] at 4 weeks postpartum, and a reduction in depressive symptoms during the early postpartum period. The results of the study developed by Liu et al. [24] for pregnant women aged 36–38 weeks were along the same lines. They demonstrated the efficacy of an app-based intervention (We’ll App), based on a combination of mindfulness and perceived social support and social support theory. The authors concluded that perceived social support can effectively reduce postpartum depressive symptoms. Similar results were obtained in the feasibility and acceptability mHealth mindfulness intervention developed by Avalos et al. [25] for women with moderate to moderately severe symptoms of postpartum depression. Participants had access to the mindfulness-based Headspace app. Women were encouraged to use the app for 10–20 min per day for 6 weeks. The authors found high rates of adherence to the intervention and acceptability and, in terms of efficacy, a decrease in symptoms of depression, stress, and improved sleep quality.

Apparently, apps help prevents health conditions by addressing their associated risk factors. Nonetheless, since zero risk does not exist in the use of health technologies, for an app to be recommended, it is essential that it is first reviewed and evaluated. Although specific criteria have not been established for the evaluation of apps, previous research has suggested that at least their content and characteristics should be considered for evaluation [26]. The purpose of previous evaluation is to guarantee the efficacy of the app and ensure it suits the needs of the final user.

Several studies have been conducted to assess these parameters in apps for the prevention of postpartum depression in other languages [16,17]. However, no studies have been conducted so far to assess the characteristics of apps available in Spanish.

Our aim was to identify which apps in Spanish available on iOS and Android could be recommended based on their content, quality, and BCTs as complementary tools to prevent postpartum depression in women.

## 2. Materials and Methods

### 2.1. Design

This is a systematic, step-by-step review that included two steps: (i) identification and selection of apps for “Postpartum depression” or apps that include topics related to postpartum depression available on iOS Store and Google Play between March and May 2023; (ii) assessment of app quality, content, and behaviour change potential.

### 2.2. Intervention

#### 2.2.1. Step 1: Selection of Smartphone Apps

Our methods sought to replicate the way a patient might access a “postpartum depression” app. Searches were performed on iOS App and Google Play Stores using the following Spanish keywords in the two stores: “embarazo” (pregnancy), “postparto” (postpartum), “depression postparto” (postpartum depression), and “prevenir la depression postparto” (preventing postpartum depression). Searches were performed using iPhone XR, iPad Pro, Oppo Find X3Pro, and Lenovo Tab M10.

Two researchers carried out an initial review of the apps available based on the description provided on the two digital stores. Duplicated apps retrieved after using different keywords and/or available in the two stores were eliminated.

The apps meeting the following criteria were included: (i) available in Spanish; (ii) free to use; (iii) targets the population under study. “Off-topic” apps were excluded. The apps meeting the criteria previously described were downloaded. The same researchers performed a second review of the final selection by considering the same inclusion and exclusion criteria. Malfunctioning apps were excluded (the screen went blank and did not advance; the app closed automatically).

The retrieved apps were considered suitable for recommendation, and their quality, content, and behaviour change potential were assessed.

#### 2.2.2. Step 2: Quality, Content and Behaviour Change Potential Assessment

Two independent reviewers assessed apps’ quality and behaviour change potential for postpartum depression.

Quality was evaluated using the Spanish version of the Mobile Application Rating Scale (MARS) (internal consistency α > 0.77) [27]. This is a tool adapted to Spanish in 2019 and has since been successfully used in health app evaluations. The Spanish MARS version includes 23 items that assess objective quality (19 items), which has four dimensions (engagement, functionality, aesthetics, and information quality), and subjective quality (4 items). All items are rated on a 5-point scale (1, inadequate; 2, poor; 3, acceptable; 4, good; 5, excellent). Total scores range from 1 (lowest objective and/or subjective quality) to 5 (highest objective and/or subjective quality). Mean scores were calculated for each item, and overall app quality was calculated by averaging the aggregated mean for all items. Behaviour change potential was evaluated using the App Behaviour Change Scale (ABACUS) [28], which includes 21 items grouped into the following 4 categories: knowledge and information (5 items), goals and planning (3 items), feedback and monitoring (7 items), and actions (6 items). A total score out of 21 was calculated by summing each item score, where a higher score suggests greater confidence in behaviour change potential.

Finally, a content analysis was performed to identify the topics addressed in each app. A researcher identified all the topics related to the purposes of our study and divided information into categories.

In addition, experts in the field were asked to provide app references. Reference lists from other relevant reviews on related subjects were manually searched.

### 2.3. Data Analysis

Total mean score and standard deviation (SD) in each domain on the MARS27 and ABACUS [28] scales and other topics were calculated. Pearson’s rank correlation was used to determine the level of agreement between MARS [27] and ABACUS [28] scores obtained by the two evaluators.

Pearson’s rank correlation was used to determine if there was any relationship between app quality, ABACUS,28 and the other topics considered.

All statistical analyses were conducted using IBM SPSS version 27.0 with significance levels at *p* value < 0.05.

## 3. Results

**Figure 1:**
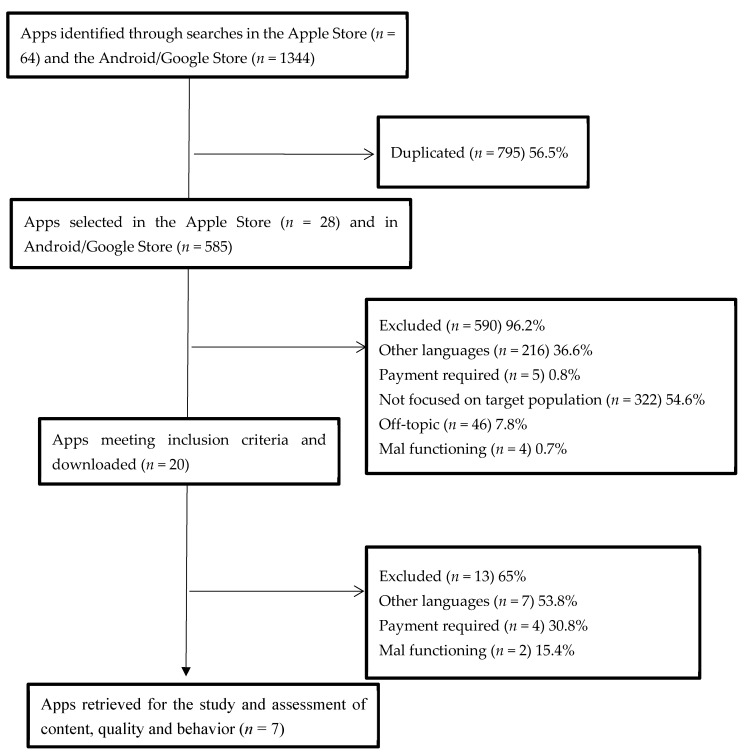
Flowchart of app search process.

A total of 1408 apps were identified. After duplicated apps were excluded (*n* = 795), 96.2% were eliminated (*n* = 590). Finally, 20 apps were downloaded, of which 65% (*n* = 13) were eliminated. The final sample was composed of seven apps that were included for quality and content evaluation (Figure 1). None of these free apps for assessing postpartum depression was available in Spanish. Therefore, all selected apps (*n* = 7) were considered because they addressed topics that could contribute to prevent postpartum depression.

### 3.1. Quality Assessment and ABACUS Score

Quality scores ranged from 1.5 to 4.1 and from 1.0 to 3.9 for objective and subjective quality, respectively. The mean accurate quality was 3.1 (SD = 1.01), whereas mean subjective quality was 2.7 (SD = 1.27) (Table 1). More than 70% of the apps had a score above 3.0 for both, objective and subjective quality (Table 1).

### 3.2. Content Assessment

A total of 24 topics were identified with a mean number of 9.9 (SD = 5.90). The most frequent topics addressed in the apps were “nutrition”, “physical activity”, “behavioral, cognitive training”, and “parental bond”. The lowest number of categories addressed in the app was 1, and the highest was 17 (Table 3).

### 3.3. Correlation between Content, MARS, and ABACUS Score

The Pearson correlation analysis showed a significant and direct association between the number of topics included in the app, objective and subjective quality, and ABACUS score (Table 4).

## 4. Discussion

The results show that a specific free postpartum depression app is not available in Spanish. Although thousands of apps are commercially available, only seven free apps were found to be recommendable for pregnant women, as they included suitable postpartum depression content, had adequate quality, and included BCTs.

The results show that the apps with the best quality scores were those that addressed a higher number of topics and BCTs. This same circumstance has been observed previously in studies carried out in Spain, in pregnancy-related apps [26]. Something similar was observed in the study developed by Musgrave et al. [16], where the Ovia Pregnancy Tracker app obtained the highest score in terms of Quality and a high number of BCTs. This finding suggests that a ranking could be established to recommend apps, with those with better quality, content, and behaviour change techniques scoring at the top. From a pragmatic point of view, this type of study not only has a scientific value but also a clinical value, since it can be considered as a guide by the healthcare personnel and can serve as an orientation in consultation.

This finding means that, according to the criteria used by our researchers, approximately 0.5% of the apps should be recommended. This result is consistent with previous research, which concludes that a low percentage of commercially available obstetrics-gynaecology apps should be recommended [29,30]. This percentage increases for Spanish obstetrics-gynaecology apps [26].

As the World Health Organization recognizes, health apps have the potential to empower people to improve their health behaviours and self-manage their health conditions [31]. Previous studies have provided empirical evidence of the effectiveness of app-based interventions in reducing postpartum depression [32]. However, the results obtained in our study highlight the need to evaluate health apps rigorously before issuing a recommendation. It is important to emphasize that zero risk does not exist in the use of apps.

The mean MARS score observed was 3.1 and 2.7 for objective and subjective quality, respectively, indicating moderate quality. In a previous systematic review and meta-analysis in which MARS was used to evaluate apps specifically designed for assessing postpartum depression and anxiety, scores ranged from 2.4 to 4.3, which also indicated moderate to high quality [33]. Similar results were obtained in previous studies evaluating apps for promoting healthy behaviours among pregnant women [26,34]. MARS provides information about app quality, based on the feasibility and effectiveness of apps [35]. This tool suggests that only high-quality apps should be recommended as, hypothetically, it will contribute to fulfil the purpose of the app.

As previous research has emphasized, incorporating behavioural theories and behaviour change techniques in digital-based interventions is highly recommended [36,37]. In this sense, other authors have suggested incorporating BCTs in apps to improve the effectiveness of digital-based interventions for promoting behaviour change [28,38].

This study revealed that apps include a limited number of BCTs. The ABACUS score obtained in our study was 6.6, which indicates that a low number of BCTs were included in the selected apps. This score is lower than reported in previous studies on apps for pregnant women. For example, the scoping review developed by Lazarevic et al. [39] showed a mean total score of 21.1% and 37.9% for knowledge and information, 21.7% for feedback and monitoring, 13.6% for actions, and 11.3% for goals and planning. In a systematic search of healthy lifestyle apps, the ABACUS average score was 7.8, ranging from 1 to 17, whereas apps for improving mental well-being had an average score of 8.7 [40].

According to previous research, more BCTs should be included in these apps to ensure their effectiveness in preventing postpartum depression. However, analytical studies should be performed to confirm this hypothesis.

Apps most commonly addressed “diet”, “physical activity”, “behavioral cognitive training”, and “parental bond”. The inclusion of these topics is relevant, since they are risk factors of postpartum depression [1,6,7,9]. To its prevention and its effects, it is well known that addressing lifestyle habits and implementing interventions aimed at improving healthy lifestyle habits during pregnancy [41], reinforcing social support, [3,11], and reducing stress [8] are essential. To this end, apps should be used as complementary healthcare tools [42]. The literature describes effective mobile-delivered interventions based on mindfulness [43], cognitive therapies [23], dietary habits [41], or exercise [44]. One aspect to highlight in relation to the content observed in the apps is that they could have positive effects not only on postpartum depression but also on other aspects such as, for example, the promotion of physical activity, decreasing the risk of excessive weight gain, or the development of a healthy diet [26].

Without a doubt, health professionals are essential during pregnancy. Midwives, for example, are still the preferred source of information for women [42], which does not exclude apps for use as complementary care tools. Previous studies have revealed that the use of apps has not only been effective in improving women’s knowledge and healthy behaviours [45,46], but it is also well accepted and has proven cost effectiveness [43].

The results of this study show that the apps with the best quality scores were those that addressed a higher number of topics and BCTs. This finding suggests that a ranking could be established to recommend apps, with those with better quality, content, and behaviour change techniques scoring at the top.

The use of apps can contribute to reducing inequalities in access to health services by mitigating the barriers that can cause them [15], and they are tools accepted by groups of women belonging to vulnerable groups [47]. There is evidence showing how certain personal or sociodemographic characteristics, such as lower educational level or worse economic situation are associated with an increased risk of mental problems during the postnatal period, as shown by the results of the study developed by Wszołek et al. [48] scored ≥10 points in Edinburgh Postnatal Depression Scale, or in other words, an increased risk of mood disorders during the postpartum period.

In the same vein, it is essential, as suggested by the results of the study conducted by Pierce et al. [48], to avoid the exclusion of app use, especially by women belonging to vulnerable groups or at identified risk of postpathologic depression, to encourage engagement. To this end, these authors recommend involving both healthcare professionals and women in the implementation of apps [49]. In the same vein, as indicated by Hughson et al. [50], in order to encourage the use of apps by pregnant women from vulnerable groups, such as those with low cultural or low-income levels, it is essential to consider their health literacy. In our opinion, health authorities must make an effort to improve health literacy if they want to implement hybrid health systems, where both phase-to-face and digital care are combined. On the other hand, according to the e-health Literacy Framework [51], it is essential that patients are motivated to use digital resources.

Finally, another aspect that has been reported in the literature is the need for greater involvement of healthcare professionals in the recommendation of apps, and, as indicated in the literature, the results of this study will help professionals to have a guide of recommended apps based on content and quality. Este factor ha sido descrito en la literatura como esencial para fomentar la prescripción de apps [52].

Some limitations of this study should be noted. We included free apps because we want to act in accordance with the characteristics of the Spanish healthcare model: free and universal. Another possible limitation is related to the absence of standardized search terms. We decided to use the most suitable terms in accordance with women in antenatal period and our researchers’ own experience and replicated the way a patient might access a postpartum depression app. Finally, this is the first study to assess the effectiveness of postpartum depression apps in Spanish, and more research is needed to further assess the effectiveness of these apps.

As strengths, the results highlight the potential of the evaluated applications for use in clinical practice. Therefore, the target audience for these apps are healthcare professionals and, indirectly, pregnant women. The adequate content and quality of the applications suggest that midwives and other health professionals could recommend them to pregnant women and consider them as a complement to usual care to prevent postpartum depression. In addition, this research provides guidance to policymakers on the shortage of scientifically supported applications to prevent postpartum depression and will inform decisions about evaluating and regulating such applications.

Finally, it is important to highlight that the results could help guide future experimental research designs aimed at evaluating the effectiveness of using apps to prevent postpartum depression, which will allow their use in clinical settings, not only based on content and quality but also on scientific criteria.

## 5. Conclusions

The results of this study show that there are no free postpartum depression apps available in Spanish, and only 0.5% free apps were found to have enough quality and adequate content to be recommended. The contents of the apps selected meet the potential needs of women and quality is adequate, but they should include a higher number of BCTs.

## Figures and Tables

**Table 1:** Mean (SD) MARS score and correlation between raters.

	Total	Rater 1	Rater 2	r (*p* Value)
Objective quality	3.1 (1.01)	3.1 (1.00)	3.1 (1.02)	0.997 (<0.001)
Engagement	3.1 (1.32)	3.1 (1.29)	3.1 (1.36)	0.990 (<0.001)
Functionality	4.3 (0.78)	4.3 (0.85)	4.3 (0.78)	0.867 (0.012)
Esthetics	3.6 (1.17)	3.4 (1.13)	3.7 (1.22)	0.970 (<0.001)
Information	2.2 (0.99)	2.2 (1.09)	2.3 (0.91)	0.978 (<0.001)
Subjective quality	2.7 (1.27)	2.8 (1.37)	2.7 (1.21)	0.948 (<0.001)

The mean ABACUS score was 6.6 (SD = 3.64). Goals obtained the lowest score, whereas knowledge got the highest (Table 2).

**Table 2:** Mean (SD) ABACUS score and correlation between raters.

	Total	Rater 1	Rater 2	r (*p* Value)
ABACUS	6.6 (3.64)	6.6 (3.64)	6.7 (3.35)	0.970 (<0.001)
Knowledge & information	3.6 (1.14)	3.4 (1.51)	3.3 (1.38)	0.970 (<0.001)
Goals and planning	0	0	0	-
Feedback and monitoring	1.8 (1.41)	1.7 (1.38)	1.9 (1.46)	0.966 (<0.001)
Actions	1.5 (1.12)	1.4 (1.40)	1.6 (0.98)	0.768 (0.044)

**Table 3:** Number of topics related to postpartum depression included in each app.

App	Number of Topics Included in the App
Baby Mam—Tu embarazo	13
BabyClub: Mi embarazo día a día	14
Mi embarazo	4
iNatal. App de embarazo	13
Dana Embarazo y Posparto	17
Laia Contigo	7
Mom & Baby	1
BabyClub: Mi embarazo día a día	14

**Table 4:** Correlation between MARS, ABACUS, and number of topics.

	Objective Quality	Subjective Quality	ABACUS	Number of Topics
Objective quality	-	0.973 **	0.926 *	0.860 *
Subjective quality	-	-	0.947 **	0.784 *
ABACUS	-	-	-	0.855 *

*p* Value * < 0.05; ** < 0.001.

## Data Availability

The data underlying this article will be shared on reasonable request to the corresponding author.

## Public Involvement Statement

No public involvement in any aspect of this research.

## Guidelines and Standards Statement

This manuscript was drafted against the guideline for reporting systematic reviews of outcome measurement instruments (OMIs): PRISMA-COSMINfor OMIs2024.

## References

[1] Agrawal I., Mehendale A.M., Malhotra R. (2022). Risk Factors of Postpartum Depression. Cureus.

[2] World Health Organization Maternal Mental Health. https://www.who.int/teams/mental-health-and-substance-use/promotion-prevention/maternal-mental-health#:~:text=Worldwide%20about%2010%25%20of%20pregnant,a%20mental%20disorder%2C%20primarily%20depression.

[3] Wang Z., Liu J., Shuai H., Cai Z., Fu X., Liu Y., Xiao X., Zhang W., Krabbendam E., Liu S. (2021). Mapping global prevalence of depression among postpartum women. Transl. Psychiatry.

[4] Míguez M.C., Vázquez M.B. (2023). Prevalence of postpartum major depression and depressive symptoms in Spanish women: A longitudinal study up to 1 year postpartum. Midwifery.

[5] Hutchens B.F., Kearney J. (2020). Risk Factors for Postpartum Depression: An Umbrella Review. J. Midwifery Women’s Health.

[6] Wu C.H., Gau M.L., Cheng S.F., Chen T.L., Wu C.J. (2023). Excessive gestational weight gain and emotional eating are positively associated with postpartum depressive symptoms among taiwanese women. BMC Women’s Health.

[7] Almasaudi A.S., Alashmali S., Baattaiah B.A., Zedan H.S., Alkhalaf M., Omran S., Alghamdi A., Khodary A. (2023). Dairy products intake and the risk of postpartum depression among mothers: A pilot study. SAGE Open Med..

[8] Wang D., Li Y.L., Qiu D., Xiao S.Y. (2021). Factors Influencing Paternal Postpartum Depression: A Systematic Review and Meta-Analysis. J. Affect. Disord..

[9] Lau E., Adams Y.J. (2023). Predictors of Postpartum Depression Among Women with Low Incomes in the United States. MCN Am. J. Matern. Child Nurs..

[10] Okun M.L., Lac A. (2023). Postpartum Insomnia and Poor Sleep Quality Are Longitudinally Predictive of Postpartum Mood Symptoms. Psychosom. Med..

[11] Kim S., Kim D.J., Lee M.S., Lee H. (2023). Association of Social Support and Postpartum Depression According to the Time After Childbirth in South Korea. Psychiatry Investig..

[12] DelRosario G.A., Chang A.C., Lee E.D. (2013). Postpartum depression: Symptoms, diagnosis, and treatment approaches. JAAPA.

[13] Ghaedrahmati M., Kazemi A., Kheirabadi G., Ebrahimi A., Bahrami M. (2017). Postpartum depression risk factors: A narrative review. J. Educ. Health Promot..

[14] Khan R., Waqas A., Bilal A., Mustehsan Z.H., Omar J., Rahman A. (2020). Association of Maternal depression with diet: A systematic review. Asian J. Psychiatr..

[15] Lewkowitz A.K., Whelan A.R., Ayala N.K., Hardi A., Stoll C., Battle C.L., Tuuli M.G., Ranney M.L., Miller E.S. (2024). The effect of digital health interventions on postpartum depression or anxiety: A systematic review and meta-analysis of randomized controlled trials. Am. J. Obstet. Gynecol..

[16] Musgrave L.M., Kizirian N.V., Homer C.S.E., Gordon A. (2020). Mobile Phone Apps in Australia for Improving Pregnancy Outcomes: Systematic Search on App Stores. JMIR mHealth uHealth.

[17] Seo J.M., Kim S.J., Na H., Kim J.H., Lee H. (2022). Effectiveness of a Mobile Application for Postpartum Depression Self-Management: Evidence from a Randomised Controlled Trial in South Korea. Healthcare.

[18] Adamo K.B., Semeniuk K., da Silva D.F., Souza S.C.S., Baillargeon J.P., Redman L.M., Piccinini-Vallis H., Shen G.X., Nerenberg K. (2023). SmartMoms Canada: An evaluation of a mobile app intervention to support a healthy pregnancy. Contemp. Clin. Trials.

[19] Daehn D., Martens C., Loew V., Kemmler L., Rudolf S., Kochen E., Renneberg B., Pawils S. (2023). SmartMoms—A web application to raise awareness and provide information on postpartum depression. BMC Pregnancy Childbirth.

[20] Varma D.S., Mualem M., Goodin A., Gurka K.K., Wen T.S., Gurka M.J., Roussos-Ross K. (2023). Acceptability of an mHealth App for Monitoring Perinatal and Postpartum Mental Health: Qualitative Study With Women and Providers. JMIR Form. Res..

[21] Miura Y., Ogawa Y., Shibata A., Kamijo K., Joko K., Aoki T. (2023). App-based interventions for the prevention of postpartum depression: A systematic review and meta-analysis. BMC Pregnancy Childbirth.

[22] Cox J.L., Holden J.M., Sagovsky R. (1987). Detection of postnatal depression. Development of the 10-item Edinburgh Postnatal Depression Scale. Br. J. Psychiatry.

[23] Qin X., Liu C., Zhu W., Chen Y., Wang Y. (2022). Preventing Postpartum Depression in the Early Postpartum Period Using an App-Based Cognitive Behavioral Therapy Program: A Pilot Randomized Controlled Study. Int. J. Environ. Res. Public Health.

[24] Liu C., Chen H., Zhou F., Long Q., Wu K., Lo L.M., Hung T.H., Liu C.Y., Chiou W.K. (2022). Positive intervention effect of mobile health application based on mindfulness and social support theory on postpartum depression symptoms of puerperae. BMC Women’s Health.

[25] Avalos L.A., Aghaee S., Kurtovich E., Quesenberry C., Nkemere L., McGinnis M.K., Kubo A.A. (2020). Mobile Health Mindfulness Intervention for Women With Moderate to Moderately Severe Postpartum Depressive Symptoms: Feasibility Study. JMIR Ment. Health.

[26] Muñoz-Mancisidor A., Martin-Payo R., Gonzalez-Mendez X., Fernández-Álvarez M.D.M. (2021). Content, Behavior Change Techniques, and Quality of Pregnancy Apps in Spain: Systematic Search on App Stores. JMIR mHealth uHealth.

[27] Martin Payo R., Álvarez M.M.F., Blanco Díaz M., Cuesta Izquierdo M., Stoyanov S.R., Llaneza Suárez E. (2019). Spanish adaptation and validation of the Mobile Application Rating Scale questionnaire. Int. J. Med. Inform..

[28] McKay F.H., Slykerman S., Dunn M. (2019). The App Behavior Change Scale: Creation of a Scale to Assess the Potential of Apps to Promote Behavior Change. JMIR mHealth uHealth.

[29] Perry R., Lunde B., Chen K.T. (2016). An evaluation of contraception mobile applications for providers of family planning services. Contraception.

[30] Salas-Wright C.P., AbiNader M.A., Vaughn M.G., Sanchez M., De La Rosa M. (2019). Trends in participation in teen pregnancy and STI prevention programming, 2002–2016. Prev. Med..

[31] Maaß L., Freye M., Pan C.C., Dassow H.H., Niess J., Jahnel T. (2022). The Definitions of Health Apps and Medical Apps From the Perspective of Public Health and Law: Qualitative Analysis of an Interdisciplinary Literature Overview. JMIR mHealth uHealth.

[32] Eisner E., Lewis S., Stockton-Powdrell C., Agass R., Whelan P., Tower C. (2022). Digital screening for postnatal depression: Mixed methods proof-of-concept study. BMC Pregnancy Childbirth.

[33] Tsai Z., Kiss A., Nadeem S., Sidhom K., Owais S., Faltyn M., Lieshout R.J.V. (2022). Evaluating the effectiveness and quality of mobile applications for perinatal depression and anxiety: A systematic review and meta-analysis. J. Affect. Disord..

[34] Hayman M., Alfrey K.L., Cannon S., Alley S., Rebar A.L., Williams S., Short C.E., Altazan A., Comardelle N., Currie S. (2021). Quality, Features, and Presence of Behavior Change Techniques in Mobile Apps Designed to Improve Physical Activity in Pregnant Women: Systematic Search and Content Analysis. JMIR mHealth uHealth.

[35] Mohan S. (2023). Usability and Quality Evaluation of the “E-Midwife” Mobile Application for Nurse-Midwives in Obstetric Complications: A Randomized Controlled Trial. Int. J. Community Based Nurs. Midwifery.

[36] Klonoff D.C. (2019). Behavioral Theory: The Missing Ingredient for Digital Health Tools to Change Behavior and Increase Adherence. J. Diabetes Sci. Technol..

[37] Taj F., Klein M.C.A., van Halteren A. (2019). Digital Health Behavior Change Technology: Bibliometric and Scoping Review of Two Decades of Research. JMIR mHealth uHealth.

[38] Thomas Craig K.J., Morgan L.C., Chen C.H., Michie S., Fusco N., Snowdon J.L., Scheufele E., Gagliardi T., Sill S. (2021). Systematic review of context-aware digital behavior change interventions to improve health. Transl. Behav. Med..

[39] Lazarevic N., Lecoq M., Bœhm C., Caillaud C. (2023). Pregnancy Apps for Self-Monitoring: Scoping Review of the Most Popular Global Apps Available in Australia. Int. J. Environ. Res. Public Health.

[40] McKay F.H., Wright A., Shill J., Stephens H., Uccellini M. (2019). Using Health and Well-Being Apps for Behavior Change: A Systematic Search and Rating of Apps. JMIR mHealth uHealth.

[41] Flor-Alemany M., Migueles J.H., Alemany-Arrebola I., Aparicio V.A., Baena-García L. (2022). Exercise, Mediterranean Diet Adherence or Both during Pregnancy to Prevent Postpartum Depression-GESTAFIT Trial Secondary Analyses. Int. J. Environ. Res. Public Health.

[42] Grimes H.A., Forster D.A., Newton M.S. (2014). Sources of information used by women during pregnancy to meet their information needs. Midwifery.

[43] Leng L.L., Yin X.C., Chan C.L.W., Ng S.M. (2023). Antenatal mobile-delivered mindfulness-based intervention to reduce perinatal depression risk and improve obstetric and neonatal outcomes: A randomized controlled trial. J. Affect. Disord..

[44] Lewis B.A., Schuver K., Dunsiger S., Samson L., Frayeh A.L., Terrell C.A., Ciccolo J.T., Fischer J., Avery M.D. (2021). Randomized trial examining the effect of exercise and wellness interventions on preventing postpartum depression and perceived stress. BMC Pregnancy Childbirth.

[45] van Dijk M.R., Koster M.P.H., Oostingh E.C., Willemsen S.P., Steegers E.A.P., Steegers-Theunissen R.P.M. (2020). A Mobile App Lifestyle Intervention to Improve Healthy Nutrition in Women Before and During Early Pregnancy: Single-Center Randomized Controlled Trial. J. Med. Internet Res..

[46] Overdijkink S.B., Velu A.V., Rosman A.N., van Beukering M.D., Kok M., Steegers-Theunissen R.P. (2018). The Usability and Effectiveness of Mobile Health Technology-Based Lifestyle and Medical Intervention Apps Supporting Health Care During Pregnancy: Systematic Review. JMIR mHealth uHealth.

[47] Leziak K., Birch E., Jackson J., Strohbach A., Niznik C., Yee L.M. (2021). Identifying Mobile Health Technology Experiences and Preferences of Low-Income Pregnant Women with Diabetes. J. Diabetes Sci. Technol..

[48] Wszołek K., Żak E., Żurawska J., Olszewska J., Pięta B., Bojar I. (2018). Influence of socio-economic factors on emotional changes during the postnatal period. Ann. Agric. Environ. Med. AAEM.

[49] Pierce P., Whitten M., Hillman S. (2023). The impact of digital healthcare on vulnerable pregnant women: A review of the use of the MyCare app in the maternity department at a central London tertiary unit. Front. Digit. Health.

[50] Hughson J.P., Daly J.O., Woodward-Kron R., Hajek J., Story D. (2018). The Rise of Pregnancy Apps and the Implications for Culturally and Linguistically Diverse Women: Narrative Review. JMIR mHealth uHealth.

[51] Norgaard O., Furstrand D., Klokker L., Karnoe A., Osborne R.H. (2015). The e-health literacy framework: A conceptual framework for characterizing e-health users and their interaction with e-health systems. Knowl. Manag. E-Learn..

[52] Alkhaldi O., McMillan B., Maddah N., Ainsworth J. (2023). Interventions Aimed at Enhancing Health Care Providers’ Behavior Toward the Prescription of Mobile Health Apps: Systematic Review. JMIR mHealth uHealth.

